# Obesity in early adulthood and physical functioning in mid-life: Investigating the mediating role of c-reactive protein

**DOI:** 10.1016/j.bbi.2022.03.008

**Published:** 2022-05

**Authors:** T. Norris, J.M. Blodgett, N.T. Rogers, M. Hamer, S.M. Pinto Pereira

**Affiliations:** a Institute of Sport, Exercise and Health, Division of Surgery and Interventional Science, Faculty of Medical Sciences, UCL, London, United Kingdom; b Centre for Diet and Activity Research, MRC Epidemiology Unit, School of Clinical Medicine, University of Cambridge, Cambridge, United Kingdom

**Keywords:** Obesity, CRP, Physical functioning, Birth cohort, Healthy aging, Life course, Mediation, Epidemiology, BMI, body mass index, CI, confidence interval, CRP, C-reactive protein, DAG, directed acyclic graph, IL-6, interleukin 6, NDE, natural direct effect, NIE, natural indirect effect, OR, odds ratio, PF, physical functioning, SEP, socioeconomic position, TE, total effect, TNF, tumour necrosis factor

## Abstract

**Introduction:**

Obesity in adulthood is associated with reduced physical functioning (PF) at older ages. However, mechanisms underpinning this association are not well understood. We investigated whether and the extent to which C-reactive protein (CRP) mediates the association between early-adult obesity and mid-life PF.

**Methods:**

We used data from 8495 participants in the 1958 British birth cohort study. Body mass index (BMI), CRP and PF were measured at 33, 45 and 50y, respectively. Poor PF was defined as the lowest (sex-specific) 10% on the Short-form 36 Physical Functioning subscale. We accounted for prospectively measured confounders in early-life (e.g., social class at birth) and in mid-adulthood (e.g., 42y comorbidities). We decomposed the total effect of early-adult obesity on mid-life PF into direct and indirect (via CRP) effects, by employing a mediation analysis based on parametric g-computation.

**Results:**

The estimated total effect of obesity at 33y on poor PF at 50y, expressed as an odds ratio (OR), was 2.41 (95% CI: 1.89, 3.08). The direct effect of obesity on poor PF (i.e., not operating via CRP), was 1.97 (95% CI: 1.51, 2.56), with an indirect effect of 1.23 (95% CI: 1.10, 1.37). As such, the proportion of the total effect which was mediated by the effect of obesity on CRP at 45y, was 23.27% (95% CI: 8.64%, 37.90%).

**Conclusion:**

Obesity in early-adulthood was associated with over twice the odds of poor PF in mid-life, with approximately 23% of the obesity effect operating via a downstream effect on CRP. As current younger generations are likely to spend greater proportions of their life course in older age and with obesity, both of which are associated with poor PF, there is an urgent need to identify mechanisms, and thus potential modifiable intermediaries, linking obesity to poor PF.

## Highlights

• Obesity at 33y resulted in more than twice the odds of poor physical functioning at 50y.
• Causal mediation models assessed the role of c-reactive protein on this relationship.
• 23% of the obesity effect on PF operated via a downstream effect on CRP.

## 1 Introduction

Life expectancy is increasing worldwide; in the last two decades alone, average life expectancy increased from 66.8 years in 2000 to 73.4 years in 2019 (WHO, 2020). In the United Kingdom there were 11.8 million individuals (18% of the total population) aged over 65 years in 2016, and this number is projected to increase to 20.4 million (26% of the population) by 2041 (Office for National Statistics, 2018). Whilst gains in average life expectancy has the potential to be a hugely valuable resource to society, there is little evidence to suggest that this increasing longevity is characterised by healthy ageing, i.e., maintaining the functional ability that enables well-being in older ages (Beard et al., 2016).

Maintaining physical functioning (PF), i.e., the ability to perform the physical activities of daily living (e.g., climbing stairs or carrying groceries), at older ages enables individuals to remain independent for longer, with positive consequences for them and their families (Government Office for Science). There are also important benefits to society, as poor PF from mid-life onwards has been associated with subsequent early retirement, lower likelihood of engaging in voluntary activities (Stafford et al., 2017) and increased healthcare utilisation (Fried and Guralnik, 1997, Melzer et al., 1999), via an increased risk of falls, injuries, and acute illnesses (Branch and Meyers, 1987, Fried and Bush, 1988, Mor et al., 1994). Moreover, poor PF from mid-life onwards is associated with increased risk of mortality (Cooper et al., 2010). Accordingly, identifying modifiable life course factors associated with poor PF is critical for developing strategies aimed at attenuating age-related declines in PF. A number of distal factors that influence PF from mid-life onwards have been identified, including physical activity (Cooper et al., 2011, Artaud et al., 2016, Stenholm et al., 2016), smoking (Parker et al., 1996) and depression (Wang et al., 2002). Of particular relevance, given the high and increasing prevalence of obesity worldwide (Abarca-Gómez et al., 2017), are findings indicating a detrimental association between obesity and subsequent PF at 50y and older (Stenholm et al., 2007, Dowd and Zajacova, 2015, Rogers et al., 2020, Pinto Pereira et al., 2020). Associations between obesity in early-adulthood and subsequent PF is of particular interest as secular trends show that during this life-stage rates of overweight/obesity begin to increase rapidly in recently-born cohorts (Johnson et al., 2015). It is also during this life-stage that peak muscular strength and function is attained (Metter et al., 1999, Hurley, 1995, Volpi et al., 2004, Dodds et al., 2014). Taken together, early-adulthood may represent a critical period in relation to subsequent PF.

While an association between obesity and subsequent PF is well documented (Stenholm et al., 2007, Dowd and Zajacova, 2015, Rogers et al., 2020, Pinto Pereira et al., 2020), support for specific obesity-related mechanisms underpinning this association is scarce. One such mechanism may involve the obesity-related state of chronic low-grade inflammation, as indicated by biomarkers such as c-reactive protein (CRP) and interleukin-6 (IL-6). While some prospective studies have suggested that the direction of causation may be inflammation-obesity (Bochud et al., 2009), a number of studies have shown that obesity is a major contributor to the variation in inflammatory markers (Greenfield et al., 2004, Park et al., 2005, Aronson et al., 2004, Holmes et al., 2014). In particular, the bi-directional Mendelian randomization study by Timpson and colleagues observed a causal effect of body mass index (BMI) on circulating CRP, but not in the reverse direction (Timpson et al., 2011). These inflammatory markers have, in turn, been postulated to lead to sarcopenia, reduced muscle strength and reduced physical functioning (Ferrucci et al., 2002, Kuo et al., 2006, Anton et al., 2013). In line with this hypothesis, Stenholm and colleagues (Stenholm et al., 2008) suggested a mediating role of CRP on the association between obesity and walking limitations (a measure of poor PF). However, the temporal nature of relationships could not be disentangled because obesity, CRP and walking limitations were measured at the same time. Moreover, the study did not account for potential confounders of the CRP-walking limitations association (e.g., comorbidities) which may have biased the estimates of associations between obesity, CRP and walking limitations.

Given current knowledge gaps and methodological limitations, we aimed to assess the causal relationships between obesity, inflammatory profiles, and PF using data collected over five decades of life from a large, nationally representative British birth cohort. Specifically, we investigated the association between obesity in early-adulthood and PF in mid-life, and whether associations were mediated by CRP.

## 2 Materials and methods

Data come from the 1958 British birth cohort study, which has been described in detail elsewhere (Power and Elliott, 2006). The study enrolled 17,638 participants at birth during one particular week in March 1958 in Scotland, Wales and England and a further 920 immigrants born in the same week. In mid-adulthood (45y) it remains broadly representative of the sample from which it was drawn (Atherton et al., 2008). The sample for analysis in the current study (N = 8495) includes participants with a valid measure of PF at 50y and with CRP values at 45y ≤ 10 mg/l (as CRP >10 mg/dL might be indicative of acute infection/inflammation (Pepys and Hirschfield, 2003, Pearson et al., 2003); see Supplementary Fig. S1 for details.

### 2.1 Ethics statement

Ethical approval was given, including at 50y by the London multi-centre Research Ethics Committee and informed consent was obtained from participants at various ages, both of which cover the secondary analyses reported here. Further details are available from the study website (Centre for Longitudinal Studies, 2022) and/or cohort profile (Power and Elliott, 2006).

### 2.2 Early-adulthood obesity (33y, exposure)

At 33y, weight and height were measured by trained interviewers using standard protocols and BMI (kg/m^2^) calculated. Obesity was defined as a BMI ≥ 30 kg/m^2^.

### 2.3 C-reactive protein (45y, mediator)

A biomedical sweep was conducted at 45y and venous blood samples were obtained in a non-fasting state. C-reactive protein (mg/l) was measured on citrated plasma by high-sensitivity nephelometric analysis of latex particles coated with CRP-monoclonal antibodies (BN ProSpec protein analyzer, Dade Behring, Marburg, Germany). CRP values that were below the detection level of the assay (0.15 mg/l were arbitrarily assigned half this value (0.075 mg/l). Full standard operating protocols for the laboratory procedures can be found on the age 44y sweep webpage (Centre for Longitudinal Studies, 2022).

### 2.4 Physical functioning (50y, outcome)

At the 50y sweep, the validated (Syddall et al., 2009, Ware et al., 1996) Physical Functioning subscale of SF-36 (Ware and Sherbourne, 1992) on limitations in physical tasks due to health was administered. As in previous work (Rogers et al., 2020, Pinto Pereira et al., 2020, Palumbo et al., 2017), poor PF was defined as the lowest, sex-specific, 10th centile of the Physical Functioning subscale, which represented a score of 55 in females and 65 in males. More details regarding the scoring of the Physical Functioning subscale can be found in Supplementary Text S1.

### 2.5 Putative confounding variables

**Fig. 1:**
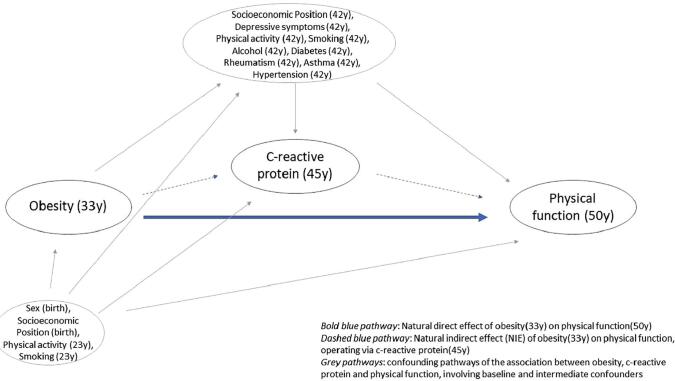
Simplified directed acyclic graph of pathways between obesity in early-adulthood and PF in mid-life.

Putative baseline and intermediate confounding variables were identified a-priori and are included in the directed acyclic graph (DAG) in Fig. 1 and Supplementary Fig. S2. Baseline confounders included sex, socioeconomic position (SEP) at birth, and physical activity and smoking status at 23y. Intermediate confounders included SEP, depressive symptoms (Rodgers et al., 1999), physical activity, smoking status, alcohol consumption, diabetes, rheumatism, asthma and hypertension (all self-reported at 42y). Details regarding the derivation, modelling and distribution of these variables can be found in Table 1 and Supplementary Table S1.

**Table 1:** Sample characteristics.

Variable (reporting age (y))	Missing (n (%))	N(%)/Median(25th, 75th centile)
Sex (birth)	–	
Male		4 106 (48.3)
Female		4 389 (51.7)

BMI (kg/m^2^) (33y)	1 529 (18.0)	24.2 (22.1, 26.9)
Male	721 (17.6)	25.0 (23.1, 27.4)
Female	808 (18.4)	23.3 (21.4, 26.2)

Obese (BMI ≥ 30 kg/m^2^) (33y)	1 529 (18.0)	700 (10.0)
Male	721 (17.6)	337 (10.0)
Female	808 (18.4)	363 (10.1)
CRP (mg/l) (45y)	2 536 (29.9)	0.9 (0.4, 2.0)
Poor PF (PF < 10th centile) (50y)	–	900 (10.6)

Social class (birth†)	239 (2.8)	
Professional/managerial		1 636 (19.8)
Skilled non-manual		855 (10.4)
Skilled manual		3 940 (47.7)
Semiskilled/unskilled manual/no male head		1 825 (22.1)

Smoking (23y)	1 272 (15.0)	
Never		3 920 (54.3)
Ex-smoker		742 (10.3)
Current		2 561 (35.5)

Physical activity (23y)	1 248 (14.7)	
Not at all in the last 4 weeks		3 646 (50.3)
1-3 times in the last 4 weeks		1 196 (16.5)
Once or twice a week		1 379 (19.0)
≥3 times a week		1 026 (14.2)

Social class (42y)	527 (6.2)	
Professional/managerial		3 103 (38.9)
Skilled non-manual		1 512 (19.0)
Skilled manual		1 285 (16.1)
Semiskilled/unskilled manual		2 068 (26.0)

Smoking (42y)	537 (6.3)	
Never		3 744 (47.1)
Ex-smoker		2 057 (25.9)
Current		2 157 (27.1)

Physical activity (42y)	540 (6.4)	
≤3 per month		2 645 (33.3)
Once per week		1 515 (19.0)
2-3 times per week		1 716 (21.6)
4-7 times per week		2 079 (26.1)

Alcohol consumption frequency (42y)	536 (6.3)	
Never		364 (4.6)
Rarely		977 (12.3)
2-4 times per month		2 390 (30.0)
At least twice per week		4 228 (53.1)
Rheumatism (42y)	584 (6.9)	358 (4.9)
Diabetes (42y)	536 (6.3)	99 (1.2)
High blood pressure (42y)	549 (6.5)	423 (5.3)
Asthma (42y)	527 (6.2)	462 (5.8)
Depressive symptoms* (42y)	583 (6.9)	2 (0, 4)
Waist-hip ratio (44y)£	1 340 (15.8)	0.9 (0.1)

† Recorded at birth or at 7y if missing at birth.

* Using 15 yes/no items from the Psychological subscale of the Malaise Inventory.

£ Summarised as mean(SD).

### 2.6 Statistical analysis

A set of preliminary regression models were developed to investigate the relationship between i) obesity at 33y and PF at 50y; ii) obesity at 33y and CRP at 45y; and iii) CRP at 45y and PF at 50y; models were initially adjusted for sex and subsequently for all other baseline confounders. Model iii was additionally adjusted for obesity at 33y. CRP was positively skewed, therefore when it was considered an outcome variable (i.e., in model ii), we transformed CRP onto the 100*log_e_ scale; regression coefficients could then be interpreted as percentage differences in CRP (i.e., sympercents (Cole, 2000). There was little evidence that associations differed by sex as tested with interaction terms (*p*_sex-interaction_ for all models ≥ 0.13), hence all analyses were sex-adjusted. To address missingness in covariate data (see Table 1), we used multiple imputation by chained equations (Royston and White, 2011), under a missing-at-random assumption and combining estimates using Rubin’s rules (Rubin, 2004). The number of imputations (n = 50) required to achieve convergence of parameter estimates was determined as 100× fraction missing information (White et al., 2011). All variables described above (i.e., obesity at 33y, CRP at 45y, PF at 50y and putative confounding variables) were included in the imputation model, as well as childhood internalizing and externalizing behaviours and cognitive ability which have been used previously in this cohort to predict missingness in follow-up (Atherton et al., 2008).

#### 2.6.1 Mediation analysis

To overcome methodological difficulties of traditional mediation approaches, including bias due to confounding between an exposure, mediator and outcome (Daniel et al., 2011), we adopted a counterfactual approach to compare hypothetical scenarios in which the entire population is obese (exposed) vs non-obese (unexposed). Specifically, to decompose the total effect (TE) of 33y obesity on the risk of 50y poor PF into a natural indirect effect (NIE; i.e., acting via 45y CRP) and a natural direct effect (NDE, i.e. not acting via 45y CRP), we used parametric g-computation using Monte Carlo simulations, performed using Stata’s ‘gformula’ command (Daniel et al., 2011). Accordingly, our targets of estimation, the total effect (TE), natural direct effect (NDE) and natural indirect effect (NIE) were estimated for the contrast obese (X = 1) vs non-obese (X = 0), with estimates for poor PF reported on the log odds scale, which were then exponentiated and presented as odds ratios (ORs) and 95% confidence intervals (CIs; calculated from bootstrapped standard errors). More details regarding the implementation of the ‘gformula’ command can be found in Supplementary Text S2. For brevity, the TE, on the log-scale, is the difference in the log-odds of poor PF in an individual with obesity (exposed, X = 1) compared to an individual without obesity (unexposed, X = 0). The NDE, on the log-scale, is the difference in the log-odds of poor PF in an individual with obesity (exposed) and with CRP (mediator) fixed to the value it would have taken had the individual not had obesity (unexposed) and the log-odds of poor PF when the individual is not obese and CRP is set to its natural non-obese value. The NIE, on the log scale, is the difference between the log-TE and log-NDE. Unlike traditional mediation approaches, the ‘gformula’ procedure is flexible, allowing for intermediate confounders between CRP and poor PF that are influenced by early adult obesity (see Fig. 1 and Supplementary Fig. S2). Statistical code and output from the g-computation procedure can be found in Supplementary Fig. S3.

All analyses were run in Stata MP v15.1.

### 2.7 Supplementary analyses

We performed three supplementary analyses to test the robustness of our findings. First, we replaced our exposure obesity, with overweight/obesity (i.e., BMI ≥ 25 kg/m^2^). Second, we re-ran analyses after reintroducing individuals with CRP >10 mg/l (n = 179) back into the sample. Finally, to identify whether and the extent to which associations may be driven by central obesity, we included waist-hip ratio at 45y as an intermediate confounder of the relationship between CRP and poor PF.

## 3 Results

### 3.1 Sample characteristics

Median BMI at 33y was 25.0 kg/m^2^ (25th, 75th centile: 23.1, 27.4) in males and 23.3 kg/m^2^ (25th, 75th centile: 21.4, 26.2) in females; approximately 10% of both males and females were classified as obese at 33y. Median CRP at 45y was 0.9 mg/l (25th, 75th centile: 0.4, 2.0) and prevalence of poor PF at 50y was (by definition) approximately 10% (Table 1). Approximately 19% of participants were ‘limited a lot’ in vigorous activities such as lifting heavy objects, while <3% were ‘limited a lot’ with respect to bathing/dressing themselves (Supplementary Table S2).

### 3.2 Association between obesity, CRP and poor PF

After adjusting for sex and baseline confounders, obesity at 33y was associated with higher CRP at 45y and poor PF at 50y (Table 2). For example, in individuals with obesity (compared to those without obesity), CRP values were 81.06% (95% CI: 70.40%, 91.72%) higher and odds of poor PF were 2.46 (95% CI: 2.01, 3.01) times greater. There was also a positive association between log_e_-CRP and odds of poor PF, with a 1-unit increase in log_e_-CRP associated with 1.40 (95% CI: 1.29, 1.53) greater odds of poor PF.

**Table 2:** Associations between obesity, CRP and poor PF (N = 8,495).

	Mean percentage difference in CRP (45 y)**				Odds ratio for Poor PF (50 y)†					
	Model A		Model B		Model A		Model B		Model C***	
	Β (95% CI)	*p- value*	Β (95% CI)	*p- value*	OR (95% CI)	*p- value*	OR (95% CI)	*p- value*	OR (95% CI)	*p- value*
Obesity at 33y *(ref: not obese)*	85.25 (74.69, 95.82)	<0.001	81.06 (70.40, 91.72)	<0.001	2.68 (2.20, 3.26)	<0.001	2.46 (2.01, 3.01)	<0.001	–	
CRP at 45y (mg/l)*	–		–		1.56 (1.44, 1.69)	<0.001	1.48 (1.37, 1.61)	<0.001	1.40 (1.29, 1.53)	<0.001

* Per unit increase on ln scale.

** Coefficients represent sympercents and are interpreted as mean percentage difference in CRP.

† Lowest, sex-specific, 10th-centile of the physical functioning subscale (female 10th centile = 55; male 10th centile = 65); Model A: adjusts for sex; Model B: Model A + adjustment for socioeconomic position at birth, smoking at 23y and physical activity at 23y; Model C: Model B + adjustment for obesity at 33y.

*** Association between obesity and poor PF was not adjusted for CRP as this would be adjusting for the mediator which would i) attenuate the total effect of obesity which is the estimand of interest here and ii) require more sophisticated mediation analyses (see statistical analysis for further details).

### 3.3 Mediation analyses

The estimated TE of obesity on poor PF is shown in Table 3, as well the decomposition of this into a NDE and NIE (via CRP) and the proportion mediated. The estimated TE of obesity at 33y on poor PF at 50y, expressed as an OR, was 2.41 (95% CI: 1.89, 3.08). The direct effect of obesity on poor PF (i.e., not operating via CRP), expressed as an OR, was 1.97 (95% CI: 1.51, 2.56); the indirect effect, via CRP, was 1.23 (95% CI: 1.10, 1.37). As such, the proportion of the total effect which was mediated by the effect of obesity on CRP at 45y, was 23.27% (95% CI: 8.64%, 37.90%).

**Table 3:** Total, natural direct and natural indirect effects (ORs (95% CI)) of obesity at 33y on poor PF at 50y (mediated by CRP at 45y).†.

	*Obesity (33y; ref: not obese)*
Total effect	2.41 (1.89, 3.08)
Natural direct effect	1.97 (1.51, 2.56)
Natural indirect effect (via CRP)	1.23 (1.10, 1,37)
Proportion mediated*	23.27 (8.64, 37.90)

† Adjusted for baseline confounders: sex, socioeconomic position at birth, physical activity at 23y, smoking at 23y, intermediate confounders: socioeconomic position, physical activity, smoking status, alcohol consumption, diabetes, rheumatism, asthma, hypertension and depressive symptoms (all at 42y).

* proportion mediated = 100*logNIE/logTE.

### 3.4 Supplementary analyses

Broadly similar patterns of associations were observed when, (i) the exposure was overweight/obesity at 33y (Supplementary Table S3), (ii) we included individuals with CRP > 10 mg/l (n = 179) in the analysis (Supplementary Table S4) and (iii) we considered waist-hip ratio at 45y as an additional intermediary confounder (Supplementary Table S5). For example, the TE of overweight/obesity on poor PF, expressed as an OR, was 1.66 (95% CI: 1.37, 2.01), with a direct effect of 1.46 (1.19, 1.78); the proportion of the total effect mediated by CRP was 26.12% (95% CI: 2.40, 49.84).

## 4 Discussion

### 4.1 Summary of findings

Using data from a large general population sample followed from birth for over five decades of life, we examined the relationship between obesity, inflammation and poor PF. To do this, we adopted a causal inference approach which appropriately accounted for relationships between, and confounders of, our variables of interest; importantly it allowed us to respect and model the temporal sequence of events. We found that individuals with obesity at 33y had over twice the odds of poor PF at 50y compared to individuals without obesity. Individuals with obesity also had CRP levels at 45y which were approximately 80% higher than individuals who were not obese. Importantly, we provide novel findings regarding the potential mediating role of inflammation on the obesity-poor PF relationship, showing that approximately 23% of the obesity effect operates via a downstream effect on CRP.

### 4.2 Strengths and limitations

Our study has a number of strengths including its large prospective design with multiple follow-up time points which enabled us to respect the temporal ordering of our exposure, mediator, outcome and confounding variables. This removes the possibility of reverse causation. Our outcome, poor PF, was assessed at approximately the same age (50y) for all individuals, thus removing the known influence of age on PF (Kuh et al., 2014). While our measure of poor PF was self-reported, it was based on the Physical Functioning subscale of the SF-36, which has been widely used in general and older populations (Ware and Sherbourne, 1992) and has been validated against objective assessments of physical performance (Syddall et al., 2009). Our definition of obesity was based on BMI, the most commonly used and widely accepted, practical measure of adiposity. However we acknowledge that, despite BMI exhibiting strong positive correlations with direct estimates of fat mass (Flegal et al., 2009), it may not adequately measure body fatness. We used the parametric g-computation procedure (‘gformula’) to model our proposed conceptual framework and this is another major study strength. This procedure allowed us to more accurately reflect processes in the real world, thus, avoiding potential biases inherent in simpler methods. For example, we were able to account for intermediate confounders such as co-morbidities in mid-adulthood (e.g., hypertension) which are themselves predicted by our exposure (i.e., obesity in early adulthood). Nonetheless, our mediation analysis relies on several assumptions including no confounding of the obesity-poor PF, obesity–CRP and CRP–poor PF associations. While availability of detailed, prospectively collected covariate data enabled us to account for several important baseline and intermediate confounders, the possibility of residual confounding cannot be ruled out. As in all longitudinal studies, loss to follow-up has occurred. Despite this, participants in mid-adulthood were broadly representative of the original population, although inevitably, the most disadvantaged were least likely to remain (Atherton et al., 2008). Hence, the possibility of selection bias must be acknowledged. Finally, as the 1958 birth cohort is predominantly of White British ethnicity (approximately 98% at 45y); we are unable to generalise results to other ethnic groups.

### 4.3 Comparison to previous studies

Previous studies examining relationships between obesity, inflammatory profiles and poor PF have been cross-sectional in nature (Stenholm et al., 2008) or examined mediation under a number of untestable and/or strict assumptions, including no confounders of the mediator-outcome association that are influenced by the exposure (Cooper et al., 2019). For example, in a cross-sectional analysis, Stenholm and colleagues, observed an attenuated association between obesity and walking limitation once CRP was accounted for (Stenholm et al., 2008). In contrast, Cooper and colleagues (Cooper et al., 2019) observed no independent effect of CRP on physical fatiguability at 68 years once BMI and IL-6 were adjusted for. Discrepancies between our and Cooper et al.’s findings could be due to differences in study design or analytic approach. Other explanations include difference in life stage examined and the ability by Cooper et al. to account for other potentially important inflammatory markers such as IL-6, which was not available in our study.

### 4.4 Interpretation of findings

We observed that 23% of the relationship between obesity in early-adulthood and PF in mid-life was mediated by CRP. An obesity-CRP-poor PF pathway is supported by the known functions and downstream effects of adipose tissue and CRP. For example, adipose tissue contributes to an increase in systemic inflammation (Berg and Scherer, 2005), via the secretion of a number of inflammatory proteins (adipocytokines) such as adiponectin, tumor necrosis factor- α (TNF-α), and IL-6 (Ouchi et al., 2011). Adipose tissue-derived IL-6 drains into the hepatic portal system and stimulates the production of CRP, an acute-phase inflammatory protein, in the liver (Yudkin et al., 2000, Mortensen, 2001, Del Giudice and Gangestad, 2018). Alternatively, obesity is associated with higher CRP via an increased mechanical load placed on joints, leading to ‘wear and tear’ (Griffin and Guilak, 2005) and increased expression of the pro-inflammatory cytokines (e.g., IL-1β and TNF-α) (Thijssen et al., 2015) which promote CRP production. At the cellular level, CRP has been associated with decreased protein synthesis rates (Toth et al., 2005), muscular degradation (Wåhlin-Larsson et al., 2017) and loss of muscle mass (Tuttle et al., 2020). It is therefore unsurprising that CRP has also been associated with reduced muscular strength (Cesari et al., 2004, Schaap et al., 2006(6):526. e9-.) and physical function (Penninx et al., 2004, Brinkley et al., 2009). We used a BMI-derived indicator of obesity to represent increased adiposity at the whole-body level. However, it is now established that the distribution of adipose tissue is an important predictor of inflammation, with centrally located visceral adipose tissue more strongly associated with markers of inflammation than subcutaneous adipose tissue (Berg and Scherer, 2005, Forouhi et al., 2001, Lapice et al., 2009). Accordingly, we suspected that the positive association between our obesity measure and CRP was likely to be driven by an increased deposition of centrally stored visceral adipose tissue. Surprisingly, when we accounted for waist-hip-ratio at 45y (a proxy for central-obesity), our estimated effects (TE, NDE and NIE) remained the same. Nonetheless, there remains a substantial effect of obesity on PF operating via pathways that do not include CRP that need to be considered. For example, obesity may lead to an accumulation of intramuscular lipids (Consitt et al., 2009), which has been associated with reduced muscular strength and physical function (Hilton et al., 2008, Tuttle et al., 2012, Marcus et al., 2012). Additionally, because BMI (and therefore obesity) tends to track over the life course (Singh et al., 2008, Norris et al., 2020a), an important pathway we have previously considered is the effect of the accumulation of a longer exposure to obesity on physical functioning (Rogers et al., 2020) and thus the mediating role of future obesity likely represents a key path linking earlier adult obesity to subsequent poor PF. In addition to the above described broadly similar patterns of associations observed when waist-hip-ratio was considered as an intermediate confounder, our findings were robust to a number of supplementary analyses, including examination of overweight and obese BMI categories together and when individuals with CRP values indicative of acute infection/inflammation (i.e., CRP >10 mg/l) were reintroduced into the sample.

### 4.5 Implications

The increasing longevity of many populations worldwide(1) means that age-related declines in PF will impact individuals for greater periods of their lives. This will occur alongside the obesity epidemic which is characterised by an increasingly earlier onset of obesity in younger generations (Johnson et al., 2015, Norris et al., 2020b). Taken together, compared to current older generations, more individuals are likely to enter early-adulthood with obesity and live longer lives with poor PF, resulting in an increased utilisation of already over-burdened healthcare systems. Thus, our finding of a potential pathway linking obesity and poor PF, via CRP, represents an important target for intervention which may reduce some of the negative consequences of obesity. Several interventions leading to reductions in CRP levels are already known (Plaisance and Grandjean, 2006). Of particular relevance is the observed positive effect of resistance training on reducing CRP levels (Sardeli et al., 2018), which as a result of its additional beneficial effects on muscular strength and PF (Chodzko-Zajko et al., 2009, Csapo and Alegre, 2016), may represent a particularly efficacious means of tackling any obesity-induced reductions in subsequent PF. Nonetheless, future studies are warranted to investigate alternative mechanisms linking obesity and poor PF, in particular the mediating roles of IL-6 and TNF-α.

## 5 Conclusions

We found that obesity in early-adulthood was associated with over twice the odds of poor PF in mid-life. We provide novel findings regarding the potential mediating role of inflammation on the obesity-poor PF relationship, with approximately 23% of the obesity effect operating via a downstream effect on CRP. As current younger generations are likely to spend greater proportions of their life course in older age and with obesity, both of which are associated with poor PF, future studies are warranted to identify further mechanisms linking obesity and poor PF.

## Declaration of Competing Interest

The authors declare that they have no known competing financial interests or personal relationships that could have appeared to influence the work reported in this paper.
