# Supplementary material for: Obesity in early adulthood and physical functioning in mid-life: Investigating the mediating role of c-reactive protein
Source: Brain Behav Immun. 2022 May;102:325–32. doi: 10.1016/j.bbi.2022.03.008 (PMC9048926; doi:10.1016/j.bbi.2022.03.008)
Supplement: Supplementary data 1 [file mmc1.docx]

**Supplementary material: Obesity in early adulthood and physical functioning in mid-life: investigating the mediating role of c-reactive protein**

Norris, T., Blodgett, J.M., Rogers, N.T., Hamer, M., Pinto Pereira, S.M

Contents

[**Supplementary Text S1: Physical Functioning subscale of the SF-36** 2](#_Toc96078315)

[**Supplementary Text S2: Mediation with ‘gformula’** 3](#_Toc96078316)

[**Supplementary Figure S1: Sample flow diagram** 5](#_Toc96078317)

[**Supplementary Figure S2: Directed acyclic graph** 6](#_Toc96078318)

[**Supplementary Table S1: Covariate specification in parametric models** 7](#_Toc96078319)

[**Supplementary Figure S3: Stata code and statistical output from the ‘gformula’ command** 8](#_Toc96078320)

[**Supplementary Table S2: Physical Functioning subscale questions: prevalence (N(%)) “limited a lot” or “limited a little”** 11](#_Toc96078321)

[**Supplementary Table S3: Total, natural direct and natural indirect effects (ORs (95% CI)) of overweight/obesity at 33y on poor PF at 50y (mediated by CRP at 45y)^†^ (N=8495)** 12](#_Toc96078322)

[**Supplementary Table S4: Total, natural direct and natural indirect effects (ORs (95% CI)) of obesity at 33y on poor PF at 50y, mediated by CRP at 45y (including individuals with CRP >10mg/l)^†^ (N=8674)** 13](#_Toc96078323)

[**Supplementary Table S5: Total, natural direct and natural indirect effects (ORs (95% CI)) of obesity at 33y on poor PF at 50y, mediated by CRP at 45y (including WHR as intermediate confounder)^†^ (N=8495)** 14](#_Toc96078324)

# **Supplementary Text S1: Physical Functioning subscale of the SF-36**

The Physical functioning subscale of the SF-36 survey(1) is a validated 10-item questionnaire measuring the extent to which individuals feel they are limited in physical tasks due to their health(1-3). The scale covers a range of severe and minor limitations, including bathing or dressing, lifting, carrying groceries, climbing stairs, bending, kneeling, stooping and walking short to moderate distances. Participants were asked to respond on a three-point scale (limited ‘a lot’, ‘a little’, ‘not at all’); scores were summed and linearly transformed to a scale ranging from 0 to 100 (lower scores represent poorer PF). Poor PF was defined as the lowest, sex-specific, 10^th^-centile of the physical functioning scale, which represented a score of 55 in females and 65 in males. Our measure of poor PF identifies those with least physical functioning at 50y and therefore at higher risk of poor functioning at older ages.

# **Supplementary Text S2: Mediation with ‘gformula’**

To estimate direct and indirect effects, the following assumptions regarding confounding are needed: i) no unmeasured confounding for the exposure-outcome relationship (conditional on covariates), ii) no unmeasured confounding for the mediator-outcome relationship (conditional on covariates and exposure), iii) no unmeasured confounding for the exposure-mediator relationship (conditional on covariates), and iv) no exposure-induced mediator-outcome confounding (conditional on covariates)(4). By using the ‘gformula’ procedure, which is based on parametric g-computation using Monte Carlo simulations, there is no need to make assumption iv, which is required when using traditional mediation approaches(5).

Standard errors were obtained via a bootstrapping procedure (with 1,000 replications) and were used to calculate 95% CIs. The number of Monte Carlo simulations was equivalent to the sample size of each model (i.e., 8495). Under a missing-at-random assumption, the ‘g-formula’ package performs a single stochastic imputation using chained equations(6). All variables in the analysis model were included in imputation models, as well as childhood internalizing and externalizing behaviours and cognitive ability which have been used previously in this cohort to predict missingness in follow-up(7).

The specified parametric models for simulation are described in Supplementary Table S1 below.

**References:**

1. Ware Jr JE, Sherbourne CD. The MOS 36-item short-form health survey (SF-36): I. Conceptual framework and item selection. Medical care. 1992:473-83.

2. McHorney CA, Ware Jr JE, Lu JR, Sherbourne CD. The MOS 36-item Short-Form Health Survey (SF-36): III. Tests of data quality, scaling assumptions, and reliability across diverse patient groups. Medical care. 1994:40-66.

3. Syddall HE, Martin HJ, Harwood RH, Cooper C, Sayer AA. The SF-36: a simple, effective measure of mobility-disability for epidemiological studies. JNHA-The Journal of Nutrition, Health and Aging. 2009;13(1):57-62.

4. VanderWeele T, Vansteelandt S. Mediation analysis with multiple mediators. Epidemiologic methods. 2014;2(1):95-115.

5. Daniel RM, De Stavola BL, Cousens SN. gformula: Estimating causal effects in the presence of time-varying confounding or mediation using the g-computation formula. The Stata Journal. 2011;11(4):479-517.

6. Van Buuren S, Boshuizen HC, Knook DL. Multiple imputation of missing blood pressure covariates in survival analysis. Statistics in medicine. 1999;18(6):681-94.

7. Atherton K, Fuller E, Shepherd P, Strachan D, Power C. Loss and representativeness in a biomedical survey at age 45 years: 1958 British birth cohort. Journal of Epidemiology & Community Health. 2008;62(3):216-23.

# **Supplementary Figure S1: Sample flow diagram**


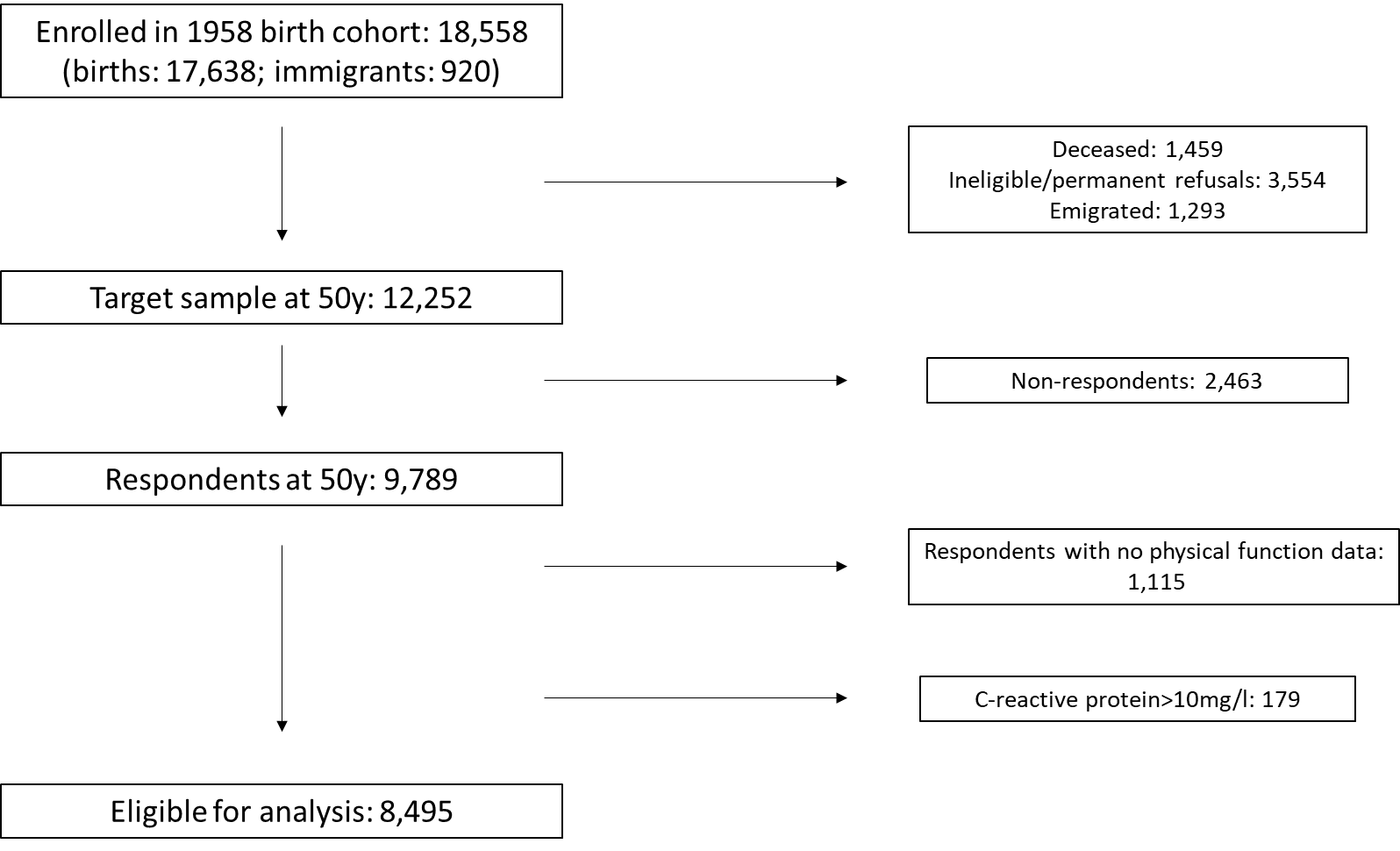


# **Supplementary Figure S2: Directed acyclic graph**


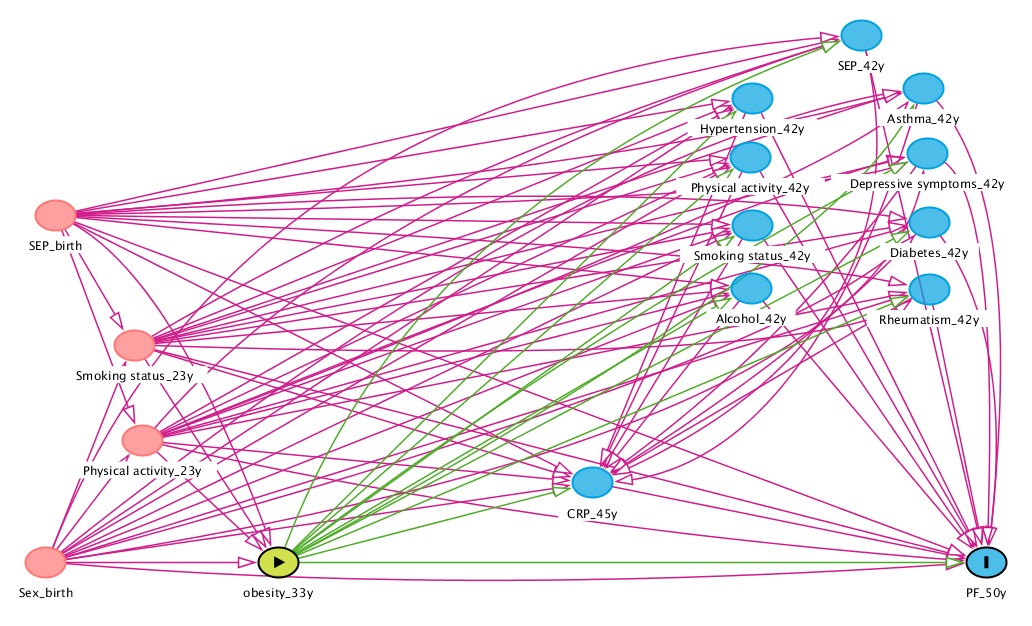


# **Supplementary Table S1: Covariate specification in parametric models**

| **Variable** | **Type of model when used as a dependent variable** | **Variables specified in parametric model** | **Functional form when used as a predictor** |
| --- | --- | --- | --- |
| *Baseline confounders* |  |  |  |
| Sex | Not predicted | - | Two categories |
| Social class (birth*) | Not predicted | - | Four categories^a^ |
| Smoking (23y) | Not predicted | - | Three categories^b^ |
| Physical activity (23y) | Not predicted | - | Four categories^c^ |
| *Post-baseline confounders* |  |  |  |
| Social class (42y) | Multinomial logistic | Sex, Social class (birth), Smoking (23y), Physical activity (23y), Obesity (33y) | Four categories^a^ |
| Smoking (42y) | Multinomial logistic | Sex, Social class (birth), Smoking (23y), Physical activity (23y), Obesity (33y) | Three categories^b^ |
| Physical activity (42y) | Multinomial logistic | Sex, Social class (birth), Smoking (23y), Physical activity (23y), Obesity (33y) | Four categories^d^ |
| Rheumatism currently (42y) | Logistic | Sex, Social class (birth), Smoking (23y), Physical activity (23y), Obesity (33y) | Two categories^e^ |
| Diabetes** (42y) | Logistic | Sex, Social class (birth), Smoking (23y), Physical activity (23y), Obesity (33y) | Two categories^e^ |
| High blood pressure** (42y) | Logistic | Sex, Social class (birth), Smoking (23y), Physical activity (23y), Obesity (33y) | Two categories^e^ |
| Asthma** (42y) | Logistic | Sex, Social class (birth), Smoking (23y), Physical activity (23y), Obesity (33y) | Two categories^e^ |
| Alcohol consumption (42y) | Multinomial logistic | Sex, Social class (birth), Smoking (23y), Physical activity (23y), Obesity (33y) | Four categories^f^ |
| Depressive symptoms (42y)^g^ | Linear | Sex, Social class (birth), Smoking (23y), Physical activity (23y), Obesity (33y) | Linear |
| *Exposure:* Obesity (33y) | Not predicted | - | Two categories^h^ |
| *Mediator:* CRP (45y) (mg/l; ln scale) | Linear | Sex, Social class (birth), Smoking (23y), Physical activity (23y), Social class (42y), Smoking (42y), Physical activity (42y), Rheumatism (42y), Diabetes (42y), High blood pressure (42y), Asthma (42y), Alcohol consumption (42y), Depressive symptoms (42y), Obesity (33y) | Linear |
| *Outcome:* Sex-specific 10^th^ centile Physical Functioning subscale, SF-36 (50y) | Logistic | Sex, Social class (birth), Smoking (23y), Physical activity (23y), Social class (42y), Smoking (42y), Physical activity (42y), Rheumatism (42y), Diabetes (42y), High blood pressure (42y), Asthma (42y), Alcohol consumption (42y), Depressive symptoms (42y), Obesity (33y), CRP (ln scale; 45y) | N/A |

^a^Social class categories (according to the Registrar General’s Classification) are: professional/managerial, skilled non-manual, skilled manual, semiskilled/unskilled manual (includes no male head at birth; includes carer/armed forces/sick/unemployed/unclassifiable at 42y);*recorded at birth or at 7y if missing at birth; ^b^Smoking categories are: never-, ex-, current-smoker; ^c^Physical activity at 23y: not at all in the last 4 weeks, 1-3 times in the last 4 weeks, once/twice a week and 3+ times a week (further details in Parsons TJ et al Med Sci Sports Exerc 2006; 38(3): 547-54); ^d^Physical activity at 42y: 4-7 times per week, 2-3 times per week, once per week, ≤3 times per month; ^e^ no/yes and based on participant self-report; ^f^Alcohol consumption: never, rarely, 2/3/4 times per month, at least twice per week; ^g^The 15-item version of Malaise inventory was used, with higher scores indicating greater psychological distress;.^h^yes/no based on measured BMI; **in last 12 months

# **Supplementary Figure S3: Stata code and statistical output from the ‘gformula’ command**

# **Supplementary Table S2: Physical Functioning subscale questions: prevalence (N(%)) “limited a lot” or “limited a little”**

|  | “limited a lot”  N(%) | “limited a little”  N(%) |
| --- | --- | --- |
| Vigorous activities, such as running, lifting heavy objects, participating in strenuous sports | 1 571 (18.5) | 3 315 (39.0) |
| Bending, kneeling or stooping | 642 (7.6) | 2 093 (24.6) |
| Walking more than one mile | 585 (6.9) | 944 (11.11) |
| Climbing several flights of stairs | 561 (6.6) | 1 530 (18.0) |
| Walking half a mile | 467 (5.5) | 520 (6.1) |
| Moderate activities, such as moving a table, pushing a vacuum cleaner, bowling, or playing golf | 434 (5.1) | 1 072 (12.6) |
| Lifting or carrying groceries | 405 (4.8) | 1 067 (12.6) |
| Climbing one flight of stairs | 323 (3.8) | 601 (7.1) |
| Walking 100 yards | 314 (3.7) | 338 (4.0) |
| Bathing or dressing yourself | 240 (2.8) | 348 (4.1) |

# **Supplementary Table S3: Total, natural direct and natural indirect effects (ORs (95% CI)) of overweight/obesity at 33y on poor PF at 50y (mediated by CRP at 45y)^†^ (N=8495)**

|  | *Overweight/obesity*  *(ref: not overweight/obese)* |
| --- | --- |
| Total effect | 1.66 (1.37, 2.01) |
| Natural direct effect | 1.46 (1.19, 1.78) |
| Natural indirect effect (via CRP) | 1.14 (1.02, 1.27) |
| Proportion mediated* | 26.12 (2.40, 49.84) |

^†adjusted for baseline confounders: sex, socioeconomic position at birth, physical activity at 23y, smoking at 23y, intermediate confounders: socioeconomic position, physical activity, smoking status, alcohol consumption, diabetes, rheumatism, asthma, hypertension and depressive symptoms (all at 42y); *proportion mediated= logNIE/logTE^

# **Supplementary Table S4: Total, natural direct and natural indirect effects (ORs (95% CI)) of obesity at 33y on poor PF at 50y, mediated by CRP at 45y (including individuals with CRP >10mg/l)^†^ (N=8674)**

|  | *Obesity (ref: not obese)* |
| --- | --- |
| Total effect | 2.48 (1.97, 3.12) |
| Natural direct effect | 1.90 (1.48, 2.44) |
| Natural indirect effect (via CRP) | 1.30 (1.17, 1.45) |
| Proportion mediated* | 29.12 (13.68, 44.55) |

^†adjusted for baseline confounders: sex, socioeconomic position at birth, physical activity at 23y, smoking at 23y, intermediate confounders: socioeconomic position, physical activity, smoking status, alcohol consumption, diabetes, rheumatism, asthma, hypertension and depressive symptoms (all at 42y); *proportion mediated= logNIE/logTE^

# **Supplementary Table S5: Total, natural direct and natural indirect effects (ORs (95% CI)) of obesity at 33y on poor PF at 50y, mediated by CRP at 45y (including WHR as intermediate confounder)^†^ (N=8495)**

|  | *Obesity (ref: not obese)* |
| --- | --- |
| Total effect | 2.44 (1.91, 3.12) |
| Natural direct effect | 1.98 (1.53, 2.55) |
| Natural indirect effect (via CRP) | 1.23 (1.10, 1.38) |
| Proportion mediated* | 23.62 (10.12, 37.11) |

^†adjusted for baseline confounders: sex, socioeconomic position at birth, physical activity at 23y, smoking at 23y, intermediate confounders: socioeconomic position, physical activity, smoking status, alcohol consumption, diabetes, rheumatism, asthma, hypertension and depressive symptoms (all at 42y) and waist-hip ratio at 45y; *proportion mediated= logNIE/log^
